# Endoportal Radiofrequency Ablation and Stent Placement in Patients with Portal Vein Tumor Thrombosis from Hepatocellular Carcinoma: A Study on Feasibility and Safety

**DOI:** 10.3390/jcm13072128

**Published:** 2024-04-07

**Authors:** Malkhaz Mizandari, Elene Gotsiridze, Pedram Keshavarz, Nariman Nezami, Tamta Azrumelashvili, Seyed Faraz Nejati, Nagy Habib, Jason Chiang, Steven S. Raman

**Affiliations:** 1 Department of Diagnostic & Interventional Radiology, New Hospitals, Tbilisi 0114, Georgia; 2 Department of Radiological Sciences, David Geffen School of Medicine, University of California, Los Angeles (UCLA), Los Angeles, CA 10833, USA; 3 School of Science and Technology, The University of Georgia, Tbilisi 0114, Georgia; 4 Department of Radiology, MedStar Georgetown University Hospital, Washington, DC 20007, USA; 5 Georgetown University School of Medicine, Washington, DC 20007, USA; 6 Lombardi Comprehensive Cancer Center, Washington, DC 20007, USA; 7 The Fischell Department of Bioengineering, University of Maryland College Park, College Park, MD 20742, USA; 8 Department of Radiology and Biomedical Imaging, Yale University School of Medicine, New Haven, CT 06510, USA; 9 Department of Surgery and Cancer, Imperial College London, London SW7 2BX, UK

**Keywords:** portal vein, tumor thrombosis, hepatocellular carcinoma, RFA, VesOpen

## Abstract

**Background**: Hepatocellular carcinoma (HCC) is the most common type of liver cancer, with 10–40% of cases involving portal vein tumor thrombosis (PVTT), leading to poor outcomes and a short survival. The effectiveness of PVTT treatment in patients with HCC is still controversial. **Methods**: This prospective dual-center study cohort comprised 60 patients with HCC and PVTT who underwent PVR-EPRFA-ST using a novel intravascular radiofrequency system followed by vascular stent placement across the PVTT stenosed segment under fluoroscopy guidance. **Results**: PVR-EPRFA-ST was technically and clinically successful in 54/60 (90%) and 37/54 (68.5%) patients, respectively. The mean tumor size, PVTT length, post-ablation luminal diameter, and median duration of the recanalized PV patency were 8.6 ± 3.4 cm, 4.1 ± 2.1 cm, 10.3 ± 1.8 mm, and 13.4 months. Higher technical and clinical success rates were associated with a longer survival (177 ± 17.3 days, HR: 0.3, 95%CI 0.12–0.71, *p* = 0.04; and 233 ± 18.3 days, HR: 0.14, 0.07–0.27, *p* < 0.001). A shorter survival was associated with Child–Pugh C (HR: 2.7, *p* = 0.04), multiple tumors (HR: 1.81, *p* = 0.03), and PVTT length (HR: 1.16, *p* = 0.04). **Conclusions**: PVR-EPRFA-ST was feasible and effective for the treatment of selected patients with PVTT, especially in patients with Child–Pugh A/B, single tumors, or a shorter PVTT length.

## 1. Introduction

Hepatocellular carcinoma (HCC) is the most common liver cancer subtype worldwide, presenting with portal vein (PV) tumor thrombosis (PVTT) in 10–40% of cases [1]. PVTT is a form of macrovascular invasion, and lobar or main PV branch involvement is associated with a poor prognosis and a short survival time [2,3]. The benefit of treating PVTT in patients with HCC remains controversial in terms of improving progression-free (PFS) and overall survival (OS) due to its underlying aggressive biology and different non-surgical therapeutic approaches with debatable benefits [4]. Intra-arterial infusion chemotherapy drugs in combination with transarterial chemoembolization (TACE) [5,6], radioembolization, and radiation therapy have been utilized for these patients with various levels of success [7]. The endoluminal probe used for endoportal radiofrequency ablation (EP-RFA) was developed for the endoscopic ablation of common bile duct (CBD) tumors and is now used for PV recanalization with stent placement (PVR-EPRFA-ST). It was adapted as a novel treatment option for HCC PVTT utilizing a percutaneously delivered endoluminal probe mated to a 480 kHz alternating-current generator, resulting in a frictional heat-based cylindrical coagulative necrosis of PVTT in a 2–3 mm radius surrounding the probe, resulting in PV recanalization [8]. EP-RFA aims to alleviate portal obstruction and enable patients to pursue additional treatments such as TACE, transarterial radioembolization (TARE), surgery, and chemotherapy [8,9,10,11,12,13,14,15]. The goal of this study was to investigate the feasibility, safety, and clinical outcomes of a combination of EP-RFA and PV stent placement in a cohort of patients with HCC and PVTT.

## 2. Materials and Methods

### 2.1. Study Design

This dual-center prospective cohort study was approved by the local institutional review board and was compliant with the United States Health Insurance Portability and Accountability Act of 1996, with written informed consent collected from each patient. From July 2012 to December 2021, a study cohort of adult patients with HCC and PVTT, verified by radiological criteria or biopsies, was treated at two interventional radiology centers—the High Technology Medical Center and the New Hospitals LTD in Tbilisi, Georgia.

### 2.2. Eligibility Criteria

Patients with PVTT from HCC invading the main PV underwent PVR-EPRFA-ST for one of the following indications: new ascites, esophageal variceal bleeding, or deterioration in liver function based on elevated AST, ALT, ALP, bilirubin, or decreased albumin and platelet counts. Patients who refused to participate in this study or who had undergone a previous minimally invasive image-guided percutaneous or transarterial treatment for HCC or presented with biliary tree dilatation or disease, active gastrointestinal bleeding, and uncorrectable coagulopathy were excluded. The final study cohort included 60 patients (54 men and 6 women; median age 57.9 ± 8.4, in a range of 46–81), 54 (90%) of whom were cirrhotic due to HCV (54/60) or HBV (6/60). Twenty-four and thirty-six patients received treatment at centers 1 and 2, respectively (Table 1).

### 2.3. Portal Vein Tumor Thrombus Imaging Diagnosis

All the patients were diagnosed with HCC and PVTT on a pre-procedure dual- or multiphase intravenous contrast-enhanced CT or MRI. Pre-procedural imaging (MRI, CT, and US) enabled appropriate patient and an appropriate patent (i.e., - free of the thrombus) peripheral intrahepatic branch PV segment selection. Regarding PVTT classification, all the patients had Vp4 PVTT based on the Japanese VP Staging System, with a patent peripheral (intrahepatic) PV branch (12). The patients were categorized into three main subcohorts by PVTT location: complete main PVTT (16.6%, 10/60), partial main with complete left PVTT (28.4%, 17/60), and partial main with complete right PVTT (55%, 33/60).

### 2.4. Portal Vein Access, Venogram, and Recanalization (PVR)

All the PVR-EPRFA-ST procedures were performed by a single interventional radiologist with 34 years of experience. The procedure was performed under conscious sedation using intravenous midazolam (1%) and sublimaze (0.005%) with local anesthesia (20 cc of 2.0% lidocaine, transcutaneous) after sterilization of the skin with betadine. The puncture site was selected based on the most obtuse angular access to a patent PV branch, with subsequent access into the PVTT segment. Using color Doppler US imaging, either the left or right PV peripheral patent branch was accessed under real-time US guidance with either a free-hand fixed-needle guide or an electromagnetic navigation technique with an 18 G trocar needle (Cook Medical, Bloomington, IN, USA) to provide optimal transhepatic access for ipsilateral PVTT processing. The approach for right PV access was subcostal or intercostal, whereas left PV puncture access was subcostal. Puncture success was validated by venous blood return via the 18 G puncture needle cannula. Under subsequent fluoroscopic guidance (at the rate of 15 frames per second), iodinated non-ionic contrast (Iohexol, Omnipaque 350; GE Healthcare, Oslo, Norway) was injected manually to confirm and document the needle tip’s adequate position in the ipsilateral PV branch. An 0.035-inch guidewire (*Roadrunner*^®^, Cook Medical Europe, Limerick, Ireland) was inserted via the 18 G needle cannula into the selected PV branch. After the guidewire’s advancement into a tributary of the punctured PV branch, an 8 Fr diameter radiopaque tip introducer sheath (*Radifocus*^®^, Terumo Europe, Leuven, Belgium) was advanced through the punctured PV branch under fluoroscopic guidance. After the introducer’s positioning, digital subtraction portography (DSP) was performed at the rate of six frames per second, opacifying PV patent peripheral branching and also showing the main PV interruption due to PVTT-induced obstruction [portography “above” the thrombus (PAT)]. Then, a 5 Fr diameter catheter (*Radifocus*^®^, Glidecath^®^, Terumo Europe, Leuven, Belgium) was introduced over the wire and manipulated down through the thrombus to the PV confluence and superior mesenteric vein (SMV). DSP was performed to image the PV segment caudally from the thrombus. DSP typically showed the interrupted main PV patency (as a vessel “amputation” or a “defect of filling”) and more or less prominent porta-portal collaterals [portography “below” the thrombus (PBT)]. Simultaneous portography “above” and “below” the thrombus (PABT) was performed to document the PVTT’s length for EP-RFA and stent implantation planning.

### 2.5. Endoportal Radiofrequency Ablation

The EP-RFA device (VesOpen, Habib) was advanced into the PVTT segment over the wire with reference to the planning portography images [8]. The EP-RFA device was mated to a commercially available RF generator (AngioDynamics 1500X; AngioDynamics Inc., Latham, NY, USA) powered at 15 W for two minutes per session, with each session covering approximately 2–2.5 cm of PVTT length.

### 2.6. Portal Vein Stent Placement

After adequate endoportal ablation of the whole length of PVTT, a 14 mm diameter self-expanding vascular stent (Zilver 635^TM^ Vascular Self-Expanding Stent, Cook Medical, Ireland) was implanted into the PV-thrombosed segment under fluoroscopic guidance, followed by catheter repositioning for control portography, which documented PV patency restoration. In cases of a tight stricture, when the self-expanding stent radial force was inadequate, balloon venoplasty was performed in order to expand the stent adequately with a 10 mm balloon (Balloon Dilator, Cook Medical, Limerick, Ireland). The recanalization was documented by final portography showing free hepatopetal flow into the liver through the formerly thrombosed portal vein.

The final portography was followed by transhepatic access tract embolization under fluoroscopic guidance using a 5 Fr diameter advantage catheter to minimize the risk of intraperitoneal bleeding. The embolization was performed by 0.035-inch diameter coil implantation (Tornado Embolization Coils; Cook Medical, Limerick, Ireland) and the injection of a Gel-Foam slurry. For coil implantation, the advantage catheter was positioned near the punctured (“target”) portal vein entry point, and the coils were advanced using a 0.035-inch diameter wire serving as a pusher. Embolization was concluded with the injection of a contrast-enhanced Gel-Foam slurry under real-time fluoroscopy guidance while the catheter was slowly withdrawn. After embolization, the puncture site was covered with a Mepore dressing. The patients were observed overnight and were discharged when clinically stable.

### 2.7. Definition of Technical and Clinical Success

**Figure 1:**
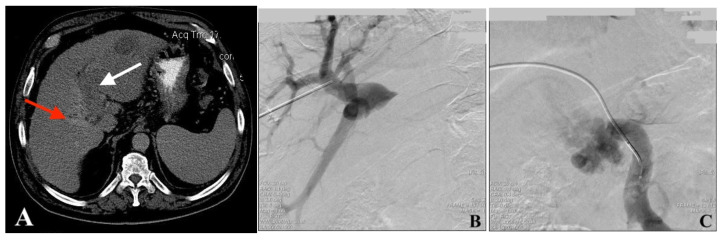

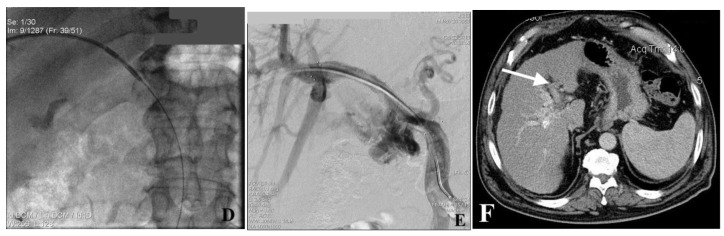
Images from a 60-year-old male patient diagnosed with HCC complicated with PVTT. (**A**) Axial CT image shows that the HCC lesion completely occupied segments 2 and 3, the left PV is obliterated by the tumor thrombus (white arrow), while the right PV remains patent (red arrow). (**B**) VesOpen procedure images in succession—the portography “above” the thrombus (PAT) shows the right PV patent branches. (**C**) VesOpen procedure images in succession—the portography “below” the thrombus (PBT) shows a dilated SMV interrupted by the tumor thrombus at the level of PV confluence; the main PV is not opacified. (**D**) Tumor thrombus processing by bipolar endoluminal VesOpen RF device. (**E**) Adequate recanalization of the PV-obstructed segment is seen after stent implantation. (**F**) Follow-up CT image (5.5 months after the procedure). The patient did not receive any other treatment. The left lobe tumor shrinkage and the not-treated left PV recanalization (white arrow) are clearly documented—abscopal effect of PV tumor thrombus RF processing.

**Figure 2:**
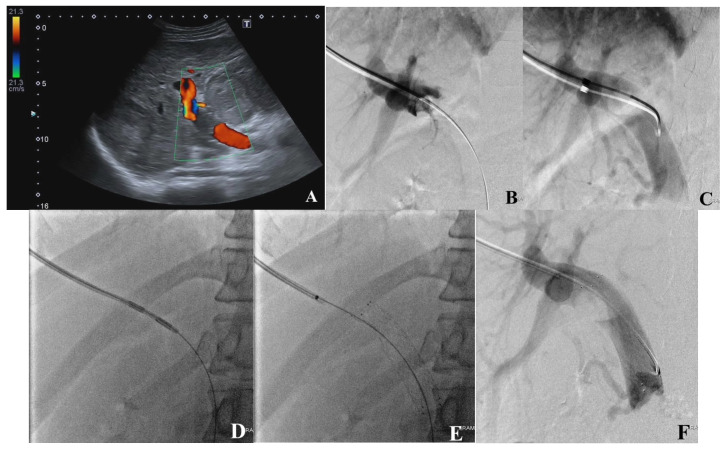
Images from a 57-year-old male patient diagnosed with HCC complicated with PVTT. (**A**) Color Doppler US depicts PV thrombus. (**B**) VesOpen procedure images in succession—wire is conducted into SMV, contrast injection via the introducer sheath depicts the oval upper surface of the tumor thrombus. (**C**) Advantage catheter is conducted into PV confluence; contrast injection depicts the main PV oval-shaped thrombus. (**D**) The tumor thrombus is processed using a bipolar endoluminal RF device. (**E**) A self-expanding vascular stent is implanted. (**F**) The portography shows PV patency restoration after the procedure.

Technical success for PVR-EPRFA-ST was defined as the recanalization of the PVTT segment with hepatopetal flow, reconnected with the main PV, or PV confluence in order to re-establish the interrupted portal blood flow into either the whole liver or lobe/segment, depending on the tumor thrombus’ location and extent. The antegrade flow after the EP-RFA procedure was documented by portal venous hepatopetal flow restoration with a decrease in portal–portal anastomosis filling in the intraprocedural DSP, performed immediately after EP-RFA and stent implantation. Clinical success was defined by the following criteria: improvement in portal hypertension and MELD score for at least six months following the procedure, improvement in clinical symptoms and liver function based on transaminase, bridging patients to transarterial embolization, and lack of tumor progression within six months of the procedure, as monitored by serial three-month US follow-ups post treatment. (Figure 1 and Figure 2).

### 2.8. Safety and Adverse Events

Adverse events (AE) of PVR-EPRFA-ST were assessed and classified into mild, moderate, severe, life-threatening, and patient death based on the Society of Interventional Radiology [SIR] classification system [16]. The patients were followed up on every two weeks for the first month post operation and then every three months.

### 2.9. Parameters Analyzed and Correlated with PVR-EPRFA-ST

The analyzed patient characteristics and clinical parameters for the assessment of PVR-EPRFA-ST feasibility and safety included patient demographics, hepatitis status, severity of cirrhosis as per the Child–Pugh score, HCC size and number (solitary vs. multiple), PVTT type (main, right, left), PVTT classification, PVTT length, post-ablation luminal diameter, pre- and post-operative laboratory changes, procedural details, AE, portal patency, and OS.

### 2.10. Statistical Analysis

The data were analyzed by SPSS (v. 18.0; IBM, SPSS Inc., Chicago, IL, USA). The log-rank test was used to determine the correlation between the variables and the survival rate, and Pearson’s chi-square test was used to compare the categorical variables. Additionally, an independent *t*-test and crosstab were used for a univariate analysis in which significant variables were included in a multivariate analysis using Cox’s proportional hazards regression. A *p* value less than 0.05 was considered statistically significant.

## 3. Results

### 3.1. Technical Success and Clinical Outcome

Six (10%, 6/60) cases had a complete technical failure due to wire conduction failure. Additionally, five (8.3%, 5/60) cases had an initial technical failure but underwent a successful PVR-EPRFA-ST on the second attempt. Primary, secondary, and overall PVR-EPRFA-ST technical success were achieved in 81.6% (49/60), 45% (5/11), and 90% (54/60) of cases, respectively. PVTT stent implantation was successfully performed in all technically successful EP-RFA cases (100%, 54/54). The mean follow-up was 5.6 months (range from 3 weeks to 23.1 months). Overall, 68.5% (37/54) of the patients had improved clinical outcomes, with significantly improved portal hypertension, MELD scores, clinical symptoms, and liver function and a lack of tumor progression within at least the first six months following the procedure. The median duration of post-treatment PV patency was 13.4 months (range from 3 weeks to 22 months).

### 3.2. Univariate and Multivariate Analyses

In a univariate analysis, we compared the survival time of the patients who achieved technical success and improved clinical outcomes with that of those who experienced a technical failure (177 ± 17.3 vs. 57 ± 38.1 days, HR: 0.3, 95%CI 0.12–0.71, *p* = 0.04) and unacceptable clinical outcomes (233 ± 18.3 vs. 56 ± 12.9 days, HR: 0.14, 95%CI 0.07–0.27, *p* < 0.001) and found that the technical and clinical success cases had a significantly longer survival time. In patients with technical success, a Child–Pugh score A/B, solitary HCC, and a PVTT ≤ 3 cm experienced an improved survival compared to the patients with Child–Pugh C (HR: 2.7, 95%CI 1.04–6.85, *p* = 0.04), multiple HCC (HR: 1.81, 95%CI 1.04–3.14, *p* = 0.03), and a PVTT > 3 cm (HR: 1.16, 95%CI 1–1.33, *p* = 0.04). Our multivariate analysis revealed that technical success (HR: 0.22, 95%CI 0.07–0.74, *p* = 0.01) and clinical outcomes’ improvement (HR: 0.12, 95%CI 0.05–0.27, *p* < 0.001) were also associated with a longer survival, while an increased HCC number was significantly negatively associated with survival time (HR: 2.8, 95%CI 1.26–6.24, *p* = 0.01). Serum laboratory values such as the tumor index alpha-fetoprotein (AFP), ALT, and AST and clinical variables such as ascites improved after the procedure, but they were not statistically significant (*p* > 0.05). Following the PVR-EPRFA-ST procedure, 31 (57%, 31/54) patients could undergo further locoregional treatments such as TACE or RFA and, in 6 cases, chemotherapy (11.1, 6/54) (Table 2).

### 3.3. Adverse Events

Using the SIR classification system [16], the AE following PVR-EPRFA-ST included fever (3.7%, 2/54), mild-to-moderate catheter positioning pain in 14.8% (8/54), some events managed conservatively (mild AE), pleural effusion, and/or abdominal hemorrhage in 5.5% of patients (3/54) (moderate AE). The post-PVR-EPRFA-ST mortality rate was 5.5% (3/54) due to thrombosis-induced liver failure, massive gastrointestinal (GI) bleeding, and intraperitoneal bleeding (patient death AE). GI bleeding resulted from rapid re-thrombosis following the procedure, while intraperitoneal bleeding occurred from the transhepatic needle track.

## 4. Discussion

In this prospective study on 60 patients with HCC-related PVTT in two different centers, percutaneous PVR-EPRFA-ST appeared to be feasible and provided a valuable therapeutic option. In almost all the cases, the PVTT was accessible for PV recanalization without AEs, using US guidance, except for one case with intraperitoneal bleeding from the tract, with successful wire and catheter transit across the PVTT in combination with portography “above” and “below” the PVTT before and after PVR-EPRFA-ST. The overall technical success of PVR-EPRFA was 90% (54/60), and the technical success of the subsequent stent placement was 100% (54/54).

EP-RFA was first reported in preclinical animal studies, demonstrating that the catheter could be used safely for vascular remodeling with increased PV flow and luminal diameter [9]. A prior pilot study using EP-RFA in six non-surgical patients with HCC-related PVTT revealed excellent safety, with immediate improvements in hepatopetal blood flow without major AEs, and suggested that the recanalization of the PV is more beneficial if combined with a therapeutic approach, such as TACE or RFA [8]. PVTT affects approximately 10% to 40% of patients with HCC [1]. Patients with HCC and PVTT are considered at an advanced stage of the disease and have a poor prognosis, with a median survival of 2.7–4 months [1,17]. The Asia–Pacific guidelines favor more aggressive surgical and locoregional therapeutic approaches, such as resection, radiotherapy, TACE, and other modalities in selected patients with HCC and PVTT [18]. A retrospective cohort study involving liver resection in patients with HCC-related PVTT reported an OS of 1.77 years, which was considerably longer than the one recorded in non-surgical approaches [19]. Another Asian multi-institutional retrospective cohort study reported that interventional approaches, including resection, had better outcomes for Child–Pugh A and selected B cirrhotic patients with Cheng’s classification PVTT type I and II, with median OS times of 15.9 and 12.5 months, respectively [20]. In Asia, surgical resection in selected patients with HCC complicated with PVTT has been reported, with acceptable short-term outcomes, although several studies reported post-operative PV thrombosis recurrence and liver failure after resection [21,22].

This study’s results showed that the EP-RFA procedure led to an improvement in liver function in around 70% of cases, accompanied by low AEs. Prior studies also reported the excellent safety of the procedure, with an increase in PV velocity after PVR-EPRFA-ST [10,11,12,13,14,15,23] (Table 3). PV stenting was reported to be safe with excellent technical success, resulting in the relief of portal obstruction with a post-procedure PV patency greater than 13 months, enabling patients to pursue additional treatments such as TACE, TARE, surgery, and chemotherapy. A retrospective study on 31 patients with HCC and PVTT divided the patients into two subgroups of PVR-EPRFA-ST vs. PVR-EPRFA without portal stenting. They revealed that EP-RFA was safe and had an excellent feasibility for patients with PVTT, without additional clinical benefit for portal vein stenting [10]. In our study, there were several advantages of using EPRFA-ST, including the partial ablation of the PVTT with partial or complete PV recanalization, decreasing portal hypertension with decreased esophageal variceal pressure, improving both the overall liver function and patients’ candidacy for palliative or potentially curative locoregional treatments.

Overall, a significant improvement in the clinical outcomes was reported in 68% of the cases, including improved symptoms of portal hypertension, liver function, and MELD score and/or a lack of tumor progression within at least the first six months after the procedure. In our univariate analysis, a Child–Pugh A/B score, a single HCC number, and a PVTT length ≤ 3 cm were independent factors of survival after percutaneous PVR-EPRFA-ST and were significantly associated with favorable outcomes. Consistent with our findings, a retrospective study observed a statistically significant association between tumor size and the prognosis of 44 HCC cases with PVTT after EP-RFA [11]. We observed that the post-procedure portography “below” the PVTT showed the recovery of the hepatopetal hepatic blood flow, leading to improved hepatic function without AEs. Therefore, EPRFA-ST might have the potential to downstage patients, allowing them to be eligible for advanced second-line therapies such as immunotherapy (e.g., Atezolizumab and Bevacizumab) or TARE, either alone or in combination with other locoregional treatments. This could also be particularly beneficial in countries with limited resources [10].

This study is primarily limited by its lack of a direct comparison between EP-RFA plus stenting and stenting alone as well as with stenting combined with other locoregional treatments. Additional limitations include the small sample size and an imbalance in the etiologies of HCC, further restricting this study’s ability to establish causality and assess treatment efficacy. Future research should aim for a more balanced inclusion of various chronic liver disease etiologies to enhance the applicability of the findings. This study also lacks comparison groups that combine recanalization with stenting and TARE to evaluate clinical success. Additionally, the availability and utilization of radioembolization in regions like Georgia and many Middle Eastern countries are hindered by high costs and other barriers, limiting the comparison with such treatments.

The overall AE rate was 5.5%, with a mortality rate of 5.5% (3/54) due to thrombosis-induced liver failure and massive variceal and intraperitoneal bleeding. Intraperitoneal bleeding may be avoided by track embolization, while left gastric vein coil embolization in the same procedure can prevent delayed esophageal bleeding. Overall, PVR-EPRFA-ST may offer some patients with HCC and PVTT a potentially improved OS if they are unsuitable for any tumor-specific treatment due to poor liver function.

## 5. Conclusions

Endoportal radiofrequency ablation with portal vein stent placement appears to be feasible and provides a valuable therapeutic option for the treatment of selected patients with hepatocellular carcinoma complicated by portal vein tumor thrombosis, although further clinical trials are required to assess its long-term safety and efficacy in a multi-institutional setting.

## Figures and Tables

**Table 1:** Baseline characteristics of patients with portal vein tumor thrombosis.

Variables	Number (%)/Mean ± SD
Age (years)	57.9 ± 8.4
Sex (male/female)	54/6
Hepatitis B	6 (10%)
Hepatitis C	54 (90%)
Cirrhotic-positive	54 (90%)
Child–Pugh classification	
A	14 (23.3%)
B	30 (50%)
C	16 (26.4%)
Tumor number (solitary/multiple)	21/39
Tumor size	8.6 ± 3.4 cm
PVTT location	
Complete main PVTT	10 (16.6%)
Partial main with left PVTT	17 (28.4%)
Partial main with right PVTT	33 (55%)
PVTT classification (Vp4 PVTT) *	60 (100%)
PVTT tumor length	4.1 ± 2.1 cm
EP-RFA procedure session	
One session	46 (76.6%)
Two sessions	11 (18.3%)
Three sessions	3 (5%)
EP-RFA procedure time	38 ± 36.4 min
Follow-up time (month)	5.6 (3 weeks to 23.1 months)

Note. PVTT: portal vein tumor thrombosis; and EP-RFA: endoportal radiofrequency ablation. * All the patients had Vp4 PVTT based on the Japanese VP Staging System, with a patent peripheral (intrahepatic) PV branch.

**Table 2:** Peri- and postoperative outcomes for patients with portal vein tumor thrombosis.

Variables	Mean ± SD (Pre/Post/Change)/Number(%)
Serology	
AST (U/L)	111.2/94.4/−16.8
ALT (U/L)	63.1/52.6/−10.5
Total bilirubin (mg/dL)	1.8/2.1/+0.3
Albumin (g/dL)	4.0/3.9/−0.1
AFP level (mg/L) *	1206(26.6, 7261)/1187(28–6943)
Ascites	54(100)/31(54.4)/−23(−42.6)
MELD score *	8(6–13)/9(7–15)
Post-procedure AE ^#^	
Mild	10(18.5%)
Moderate	3(5.5%)
Severe	0
Life-threatening	0
Patient death	3(5.5%)
Length of stay (days)	1.4 ± 0.8
Post-EP-RFA luminal diameter	10.3 ± 1.8 mm
Duration of the recanalized PV	13.4 months (3 weeks to 22 months)

Note. * Reported based on median, range; and AE: adverse events. ^#^ AE were reported based on the Society of Interventional Radiology [SIR] classification system. mRECIST: modified Response Evaluation Criteria in Solid Tumors.

**Table 3:** Review of previous documents reporting percutaneous EP-RFA using the Habib^TM^ VesOpen catheter.

First Author, Year, Country	Study Type (No. of Cases)	Population	Summary of Results
Mizandari 2013, Georgia [8]	Technical note (*n* = 6)	HCC with PVTT	- Procedure outcome: Acceptable technical success - Partial recanalization of portal vein: Safe - Major complications: None
GE 2014, China [23]	Original research (*n* = 15)	HCC with PVTT	- Procedure outcome: Acceptable - Tumor thrombosis shrunk, and PV blood flow increased - Adverse effects: Temporary pain at puncture site, transient rise in transaminases
Zhang 2015, China [12]	Experimental study (*n* = 10)	HCC with PVTT (miniature pig models)	- Procedure outcome: Acceptable - Vital consideration: Proper RF ablation time/power selection to prevent PV perforation, tissue damage
Mizandari 2016, Georgia [13]	Case report (*n* = 1)	HCC with PVTT	- Procedure outcome: Effective therapeutic approach - Well tolerated by the patient, significant tumor size decrease - No major post-procedural complications
Wu 2016, China [10]	Clinical investigation (*n* = 31)	HCC with malignant PV obstruction	- Procedure outcome: Acceptable and safe - Patient clinical symptoms: Improvement rate: 87.1% (27/31) - Procedural complications: No abdominal hemorrhage or related complications
Li 2016, China [14]	Original research (*n* = 13)	HCC with PVTT	- Procedure outcome: Technically successful - Potential advantage: Possibly better than endovascular RFA alone - Outcome risk: Tumor thrombosis progression - Complication: Liver failure
Chen 2018, China [11]	Original research (*n* = 44)	HCC with PVTT	- Procedure outcome: Clinically effective and safe - Major complications: Absent
Ding 2018, China [15]	*JVIR* abstract (*n* = 10)	HCC with PVTT	- Procedure outcome: Acceptable - Alternative therapeutic approach for HCC cases with PVTT - Complication: Absence of technique-specific complications such as hemorrhage, vessel perforation, and infection

Note. HCC: hepatocellular carcinoma; PV: portal vein; PVTT: portal vein tumor thrombosis; and *JVIR*: *Journal of Vascular & Interventional Radiology*.

## Data Availability

The data presented in this study are available on request from the corresponding authors.

## References

[1] Lu J., Zhang X.-P., Zhong B.-Y., Lau W.Y., Madoff D.C., Davidson J.C., Qi X., Cheng S.-Q., Teng G.-J. (2019). Management of patients with hepatocellular carcinoma and portal vein tumour thrombosis: Comparing east and west. Lancet Gastroenterol. Hepatol..

[2] Qadan M., Kothary N., Sangro B., Palta M. (2020). The treatment of hepatocellular carcinoma with portal vein tumor thrombosis. Am. Soc. Clin. Oncol. Educ. Book.

[3] Zhang Z.-M., Lai E.C.H., Zhang C., Yu H.-W., Liu Z., Wan B.-J., Liu L.-M., Tian Z.-H., Deng H., Sun Q.-H. (2015). The strategies for treating primary hepatocellular carcinoma with portal vein tumor thrombus. Int. J. Surg..

[4] Wang L., Guo X., Xu X., De Stefano V., Plessier A., Noronha Ferreira C., Qi X. (2021). Anticoagulation favors thrombus recanalization and survival in patients with liver cirrhosis and portal vein thrombosis: Results of a meta-analysis. Adv. Ther..

[5] Zheng K., Zhu X., Fu S., Cao G., Li W.-Q., Xu L., Chen H., Wu D., Yang R., Wang K. (2022). Sorafenib plus hepatic arterial infusion chemotherapy versus sorafenib for hepatocellular carcinoma with major portal vein tumor thrombosis: A randomized trial. Radiology.

[6] Hu J., Bao Q., Cao G., Zhu X., Yang R., Ji X., Xu L., Zheng K., Li W., Xing B. (2020). Hepatic arterial infusion chemotherapy using oxaliplatin plus 5-fluorouracil versus transarterial chemoembolization/embolization for the treatment of advanced hepatocellular carcinoma with major portal vein tumor thrombosis. CardioVascular Interv. Radiol..

[7] Chow P.K.H., Gandhi M., Tan S.-B., Khin M.W., Khasbazar A., Ong J., Choo S.P., Cheow P.C., Chotipanich C., Lim K. (2018). SIRveNIB: Selective internal radiation therapy versus sorafenib in Asia-Pacific patients with hepatocellular carcinoma. J. Clin. Oncol..

[8] Mizandari M., Ao G., Zhang Y., Feng X., Shen Q., Chen M., Lau W., Nicholls J., Jiao L., Habib N. (2013). Novel percutaneous radiofrequency ablation of portal vein tumor thrombus: Safety and feasibility. Cardiovasc. Interv. Radiol..

[9] Lazoura O., Zacharoulis D., Kanavou T., Rountas C., Katsimboulas M., Tzovaras G., Habib N. (2011). A novel experimental animal model of arterial stenosis based on endovascular radiofrequency energy application. J. Investig. Surg..

[10] Wu T.T., Li H.C., Zheng F., Ao G.K., Lin H., Li W.M. (2016). Percutaneous endovascular radiofrequency ablation for malignant portal obstruction: An initial clinical experience. Cardiovasc. Interv. Radiol..

[11] Chen Z.W., Lin Z.Y., Chen Y.P., Chen J., Chen J. (2018). Clinical efficacy of endovascular radiofrequency ablation in the treatment of portal vein tumor thrombus of primary hepatocellular carcinoma. J. Cancer Res. Ther..

[12] Zhang L., Fu J., Song P., Yuan K., Yan J., Duan F., Wang M., Liu F. (2015). The safety of Habib VesOpen bipolar radiofrequency ablation catheter used in the treatment of portal vein tumor thrombus: An experimental study in miniature pig models. J. Interv. Radiol..

[13] Mizandari M., Azrumelashvili T., Paksashvili N., Kikodze N., Pantsulaia I., Janikashvili N., Chikovani T. (2016). Tumor regression in HCC patient with portal vein tumor thrombosis after intraportal radiofrequency thermal ablation. Case Rep. Hepatol..

[14] Honglu L., Changqing L., Jiang G., Dong Z., Jian W., Liang C., Youjia D., Xiaopu H., Zhengguan C. (2016). Endovascular radiofrequency ablation of portal vein tumor thrombus with Habib TM VesOpen catheter combined with covered-stent placement: A pilot clinical study. J. Diagn. Imaging Interv. Radiol..

[15] Ding W., Wang W. (2018). Abstract No. 493 Percutaneous endovascular radiofrequency ablation for portal vein tumor thrombosis in patients with hepatocellular carcinoma: A single-center experience. J. Vasc. Interv. Radiol..

[16] Baerlocher M.O., Nikolic B., Sze D.Y. (2023). Adverse event classification: Clarification and validation of the Society of Interventional Radiology specialty–specific system. J. Vasc. Interv. Radiol..

[17] Li X., Ye Z., Lin S., Pang H. (2021). Predictive factors for survival following stereotactic body radiotherapy for hepatocellular carcinoma with portal vein tumour thrombosis and construction of a nomogram. BMC Cancer.

[18] Kudo M., Kitano M., Sakurai T., Nishida N. (2015). General rules for the clinical and pathological study of primary liver cancer, nationwide follow-up survey and clinical practice guidelines: The outstanding achievements of the Liver Cancer Study Group of Japan. Dig. Dis..

[19] Kokudo T., Hasegawa K., Matsuyama Y., Takayama T., Izumi N., Kadoya M., Kudo M., Ku Y., Sakamoto M., Nakashima O. (2016). Survival benefit of liver resection for hepatocellular carcinoma associated with portal vein invasion. J. Hepatol..

[20] Wang K., Guo W.X., Chen M.S., Mao Y.L., Sun B.C., Shi J., Zhang Y.J., Meng Y., Yang Y.F., Cong W.M. (2016). Multimodality treatment for hepatocellular carcinoma with portal vein tumor thrombus: A large-scale, multicenter, propensity mathching score analysis. Medicine.

[21] Zhang X.-P., Gao Y.-Z., Chen Z.-H., Wang K., Cheng Y.-Q., Guo W.-X., Shi J., Zhong C.-Q., Zhang F., Cheng S.-Q. (2019). In-hospital mortality after surgical resection in hepatocellular carcinoma patients with portal vein tumor thrombus. J. Cancer.

[22] Roayaie S., Jibara G., Taouli B., Schwartz M. (2013). Resection of hepatocellular carcinoma with macroscopic vascular invasion. Ann. Surg. Oncol..

[23] Ge N., Yang Y., Shen S., Yu X., Zhang Y., Wu L., Liang J., Zhu J., Cheng S., Shen F. (2014). Percutaneous radiofrequency ablation for the treatment of portal vein tumor thrombus: Experience of 15 cases. J. Interv. Radiol..

